# 3,3′-Dimethyl-1,1′-[2,2′-bipyridine-5,5′-diylbis(methyl­ene)]diimidazol-3-ium bis­(hexa­fluoro­phosphate)

**DOI:** 10.1107/S160053681103340X

**Published:** 2011-08-27

**Authors:** Sunhong Park, Suk-Hee Moon, Tae Ho Kim, Ki-Min Park

**Affiliations:** a Department of Chemistry and Research Institute of Natural Sciences, Gyeongsang National University, Jinju 660-701, Republic of Korea; b Department of Food & Nutrition, Kyungnam College of Information and Technology, Busan 617-701, Republic of Korea

## Abstract

The title compound, C_20_H_22_N_6_^2+^·2PF_6_^−^, was prepared by the reaction of 5,5′-bis­(bromo­meth­yl)-2,2′-bipyridine with 1-methyl­imidazole. The main mol­ecule lies on an inversion center located at the mid-point of the C—C bond joining the two pyridine rings. The asymmetric unit therefore contains one half-mol­ecule and one hexa­fluoro­phosphate anion. The dihedral angle between the pyridine and imidazole rings is 76.93 (7)°. In the crystal, weak inter­molecular C—H⋯F hydrogen bonds contribute to the stabilization of the packing.

## Related literature

For related syntheses, see: Sambrook *et al.* (2006 ▶); Zang *et al.* (2010 ▶). For related structures, see: Moon *et al.* (2011 ▶); Zang *et al.* (2010 ▶). For reference bond lengths, see: Allen *et al.* (1987 ▶).
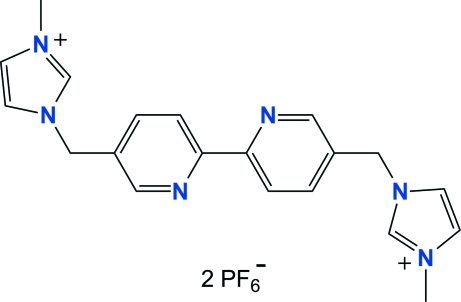


## Experimental

### 

#### Crystal data

C_20_H_22_N_6_^2+^·2PF_6_^−^
*M* *_r_* = 636.38
Monoclinic, 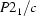

*a* = 7.5323 (4) Å
*b* = 10.7169 (6) Å
*c* = 15.4602 (9) Å
β = 93.922 (1)°
*V* = 1245.07 (12) Å^3^
*Z* = 2
Mo *K*α radiation
μ = 0.29 mm^−1^
*T* = 173 K
0.40 × 0.40 × 0.10 mm

#### Data collection

Bruker APEXII CCD diffractometer
7489 measured reflections
2717 independent reflections
1788 reflections with *I* > 2σ(*I*)
*R*_int_ = 0.049

#### Refinement

*R*[*F* ^2^ > 2σ(*F* ^2^)] = 0.040
*wR*(*F* ^2^) = 0.117
*S* = 1.02
2717 reflections
181 parameters
H-atom parameters constrained
Δρ_max_ = 0.30 e Å^−3^
Δρ_min_ = −0.29 e Å^−3^

### 

Data collection: *APEX2* (Bruker, 2006 ▶); cell refinement: *SAINT* (Bruker, 2006 ▶); data reduction: *SAINT*; program(s) used to solve structure: *SHELXTL* (Sheldrick, 2008 ▶); program(s) used to refine structure: *SHELXTL*; molecular graphics: *SHELXTL* and *DIAMOND* (Brandenburg, 1998 ▶); software used to prepare material for publication: *SHELXTL*.

## Supplementary Material

Crystal structure: contains datablock(s) global, I. DOI: 10.1107/S160053681103340X/lx2201sup1.cif

Structure factors: contains datablock(s) I. DOI: 10.1107/S160053681103340X/lx2201Isup2.hkl

Supplementary material file. DOI: 10.1107/S160053681103340X/lx2201Isup3.cml

Additional supplementary materials:  crystallographic information; 3D view; checkCIF report

## Figures and Tables

**Table 1:** Hydrogen-bond geometry (Å, °)

*D*—H⋯*A*	*D*—H	H⋯*A*	*D*⋯*A*	*D*—H⋯*A*
C7—H7⋯F1	0.95	2.23	3.111 (3)	154
C7—H7⋯F4	0.95	2.39	3.230 (3)	147
C8—H8⋯F1^i^	0.95	2.50	3.163 (3)	127
C8—H8⋯F2^i^	0.95	2.50	3.446 (3)	176
C9—H9⋯F2^ii^	0.95	2.52	3.240 (3)	133

Symmetry codes: (i) 
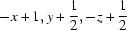
; (ii) 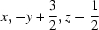.

**Fig. 1.**
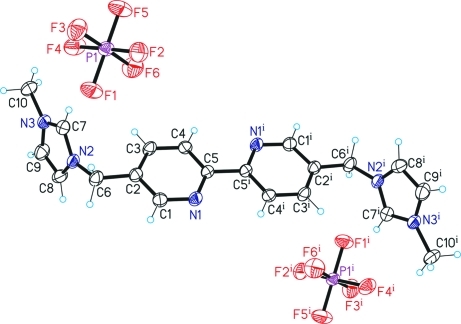
The molecular structure of the title compound, showing displacement ellipsoids drawn at the 50% probability level. Hydrogen bonds are shown as dashed lines. (Symmetry code: i) -x + 1, -y + 2, -z + 1)

**Fig. 2.**
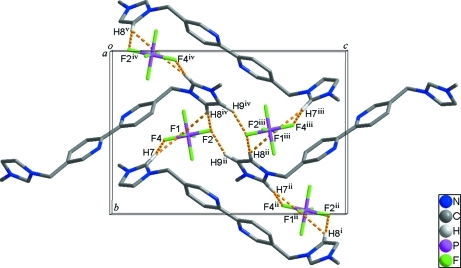
Crystal packing of the title compound with intermolecular C—H···F hydrogen bonds shown as dashed lines. (Symmetry codes: i) -x + 1, -y + 2, -z + 1; ii) x, -y + 3/2, z + 1/2; iii) -x + 1, -y + 1, -z + 1; iv) -x + 1, y - 1/2, -z + 1/2; v) x, y - 1, z).

## supplementary crystallographic information

### Comment

The title compound was prepared for use as a *N*-heterocyclic carbene ligand in the formation of coordination polymers in line with similar previously reported compounds (Sambrook *et al.*, 2006; Zang *et al.*, 2010).

In the title compound (Scheme 1, Fig. 1), two pyridine rings are coplanar because the title compound lies on a crystallographic inversion center. The geomeries of the title compound are very similar with those of the previously reported compound (Moon *et al.*, 2011) The dihedral angle between the pyridine and imidazole rings is 76.93 (7)°. All the bond lengths are within normal values (Allen *et al.*, 1987).

The crystal packing (Fig. 2) is stabilized by weak intermolecular C—H···F hydrogen bonds (see, Table 1)

### Experimental

A mixture of 1-methylimidazole (0.150 g, 1.83 mmol) and 5,5'-bis(bromomethyl)-2,2'-bipyridine (0.30 g, 0.88 mmol) in 1,4-dioxane (15 ml) was stirred for 10 min and then heated at reflux for 6 h. After cooling to room temperature, Et_2_O (15 ml) was added and 5,5'-bis((*N*-methylimidazolium-1-yl)methyl)-2,2'-bipyridine bis(chloride) obtained as a white precipitate was separated by filtration and washed with Et_2_O. For the anion exchange, an excess of KPF_6_ was added to the aqueous solution of the chloride salts. After stirring for 1 hr, the title compound as a white precipitate was obtained. X–ray quality single crystals were obtained by slow evaporation of a solution of the title compound in acetonitrile at room temperature.

### Refinement

All H-atoms were positioned geometrically and refined using a riding model with d(C—H) = 0.95 Å, *U*_iso_ = 1.2*U*_eq_(C) for aromatic, d(C—H) = 0.99 Å, *U*_iso_ = 1.2<U>_eq_(C) for methylene, and d(C—H) = 0.98 Å, *U*_iso_(H) = 1.5*U*_eq_(C) for methyl protons.

### Crystal data

**Table:**

C_20_H_22_N_6_^2+^·2PF_6_^−^	*F*(000) = 644
*M**_r_* = 636.38	*D*_x_ = 1.697 Mg m^−^^3^
Monoclinic, *P*2_1_/*c*	Mo *K*α radiation, λ = 0.71073 Å
Hall symbol: -P 2ybc	Cell parameters from 2214 reflections
*a* = 7.5323 (4) Å	θ = 2.3–27.5°
*b* = 10.7169 (6) Å	µ = 0.29 mm^−^^1^
*c* = 15.4602 (9) Å	*T* = 173 K
β = 93.922 (1)°	Plate, colorless
*V* = 1245.07 (12) Å^3^	0.40 × 0.40 × 0.10 mm
*Z* = 2	

### Data collection

**Table:**

Bruker APEXII CCD diffractometer	1788 reflections with *I* > 2σ(*I*)
Radiation source: fine-focus sealed tube	*R*_int_ = 0.049
graphite	θ_max_ = 27.0°, θ_min_ = 2.6°
φ and ω scans	*h* = −9→9
7489 measured reflections	*k* = −13→11
2717 independent reflections	*l* = −19→17

### Refinement

**Table:**

Refinement on *F*^2^	Primary atom site location: structure-invariant direct methods
Least-squares matrix: full	Secondary atom site location: difference Fourier map
*R*[*F*^2^ > 2σ(*F*^2^)] = 0.040	Hydrogen site location: inferred from neighbouring sites
*wR*(*F*^2^) = 0.117	H-atom parameters constrained
*S* = 1.02	*w* = 1/[σ^2^(*F*_o_^2^) + (0.0536*P*)^2^ + 0.3234*P*] where *P* = (*F*_o_^2^ + 2*F*_c_^2^)/3
2717 reflections	(Δ/σ)_max_ < 0.001
181 parameters	Δρ_max_ = 0.30 e Å^−^^3^
0 restraints	Δρ_min_ = −0.29 e Å^−^^3^

### Special details

**Table:**

Geometry. All e.s.d.'s (except the e.s.d. in the dihedral angle between two l.s. planes) are estimated using the full covariance matrix. The cell e.s.d.'s are taken into account individually in the estimation of e.s.d.'s in distances, angles and torsion angles; correlations between e.s.d.'s in cell parameters are only used when they are defined by crystal symmetry. An approximate (isotropic) treatment of cell e.s.d.'s is used for estimating e.s.d.'s involving l.s. planes.
Refinement. Refinement of *F*^2^ against ALL reflections. The weighted *R*-factor *wR* and goodness of fit *S* are based on *F*^2^, conventional *R*-factors *R* are based on *F*, with *F* set to zero for negative *F*^2^. The threshold expression of *F*^2^ > σ(*F*^2^) is used only for calculating *R*-factors(gt) *etc*. and is not relevant to the choice of reflections for refinement. *R*-factors based on *F*^2^ are statistically about twice as large as those based on *F*, and *R*- factors based on ALL data will be even larger.

### Fractional atomic coordinates and isotropic or equivalent isotropic displacement parameters (Å^2^)

**Table:**

	*x*	*y*	*z*	*U*_iso_*/*U*_eq_	
N1	0.6669 (2)	0.90672 (19)	0.45131 (12)	0.0314 (5)	
N2	0.4666 (2)	0.75139 (19)	0.16354 (12)	0.0292 (5)	
N3	0.2383 (3)	0.7410 (2)	0.07124 (12)	0.0327 (5)	
C1	0.6823 (3)	0.8369 (3)	0.38058 (15)	0.0335 (6)	
H1	0.7937	0.7979	0.3739	0.040*	
C2	0.5479 (3)	0.8172 (2)	0.31630 (14)	0.0272 (5)	
C3	0.3880 (3)	0.8772 (2)	0.32510 (14)	0.0292 (5)	
H3	0.2924	0.8675	0.2823	0.035*	
C4	0.3687 (3)	0.9517 (2)	0.39729 (14)	0.0272 (5)	
H4	0.2605	0.9952	0.4037	0.033*	
C5	0.5085 (3)	0.9623 (2)	0.45999 (13)	0.0232 (5)	
C6	0.5823 (3)	0.7291 (2)	0.24282 (15)	0.0328 (6)	
H6A	0.7080	0.7377	0.2288	0.039*	
H6B	0.5647	0.6423	0.2624	0.039*	
C7	0.3074 (3)	0.7000 (2)	0.14706 (15)	0.0327 (6)	
H7	0.2519	0.6430	0.1838	0.039*	
C8	0.5004 (3)	0.8271 (3)	0.09592 (16)	0.0365 (6)	
H8	0.6048	0.8755	0.0905	0.044*	
C9	0.3580 (3)	0.8208 (3)	0.03775 (16)	0.0393 (6)	
H9	0.3437	0.8636	−0.0161	0.047*	
C10	0.0635 (3)	0.7051 (3)	0.03147 (18)	0.0451 (7)	
H10A	0.0059	0.6473	0.0699	0.068*	
H10B	0.0783	0.6643	−0.0243	0.068*	
H10C	−0.0106	0.7797	0.0222	0.068*	
P1	0.04237 (8)	0.51966 (6)	0.32208 (4)	0.03076 (19)	
F1	0.24565 (19)	0.51442 (16)	0.29682 (10)	0.0478 (4)	
F2	0.1084 (2)	0.48970 (16)	0.42008 (9)	0.0504 (4)	
F3	0.0232 (2)	0.37450 (15)	0.30333 (11)	0.0547 (5)	
F4	−0.0194 (2)	0.54954 (16)	0.22308 (10)	0.0536 (5)	
F5	−0.1585 (2)	0.52519 (18)	0.34678 (11)	0.0563 (5)	
F6	0.0658 (2)	0.66508 (15)	0.33975 (12)	0.0586 (5)	

### Atomic displacement parameters (Å^2^)

**Table:**

	*U*^11^	*U*^22^	*U*^33^	*U*^12^	*U*^13^	*U*^23^
N1	0.0241 (10)	0.0412 (13)	0.0278 (11)	0.0060 (9)	−0.0061 (8)	−0.0057 (9)
N2	0.0271 (10)	0.0340 (12)	0.0258 (11)	0.0016 (8)	−0.0040 (8)	−0.0073 (8)
N3	0.0268 (11)	0.0425 (13)	0.0279 (11)	0.0007 (9)	−0.0040 (8)	−0.0065 (9)
C1	0.0228 (12)	0.0455 (16)	0.0314 (13)	0.0080 (11)	−0.0035 (10)	−0.0055 (11)
C2	0.0273 (12)	0.0298 (13)	0.0238 (12)	0.0005 (10)	−0.0024 (9)	0.0013 (10)
C3	0.0263 (12)	0.0341 (14)	0.0256 (12)	0.0019 (10)	−0.0084 (10)	−0.0010 (10)
C4	0.0217 (11)	0.0335 (14)	0.0259 (12)	0.0044 (10)	−0.0024 (9)	0.0021 (10)
C5	0.0247 (11)	0.0234 (13)	0.0210 (11)	−0.0002 (9)	−0.0021 (9)	0.0041 (9)
C6	0.0274 (13)	0.0389 (15)	0.0305 (13)	0.0065 (11)	−0.0088 (10)	−0.0061 (11)
C7	0.0319 (13)	0.0380 (15)	0.0279 (13)	−0.0030 (11)	0.0006 (10)	−0.0031 (11)
C8	0.0315 (13)	0.0407 (16)	0.0369 (14)	−0.0113 (11)	−0.0010 (11)	−0.0016 (11)
C9	0.0456 (16)	0.0418 (17)	0.0294 (14)	−0.0044 (12)	−0.0044 (11)	0.0009 (11)
C10	0.0281 (14)	0.063 (2)	0.0428 (16)	−0.0043 (13)	−0.0095 (12)	−0.0115 (14)
P1	0.0257 (3)	0.0360 (4)	0.0301 (4)	0.0002 (3)	−0.0017 (3)	0.0030 (3)
F1	0.0296 (8)	0.0637 (11)	0.0504 (10)	0.0063 (7)	0.0055 (7)	0.0140 (8)
F2	0.0494 (10)	0.0695 (12)	0.0314 (9)	−0.0041 (8)	−0.0042 (7)	0.0066 (7)
F3	0.0684 (11)	0.0394 (10)	0.0561 (10)	−0.0069 (8)	0.0020 (9)	−0.0042 (7)
F4	0.0424 (9)	0.0776 (13)	0.0390 (10)	−0.0098 (8)	−0.0112 (7)	0.0184 (8)
F5	0.0312 (9)	0.0749 (13)	0.0637 (12)	0.0039 (8)	0.0106 (8)	0.0159 (9)
F6	0.0639 (11)	0.0363 (10)	0.0760 (12)	−0.0022 (8)	0.0072 (9)	−0.0034 (8)

### Geometric parameters (Å, °)

**Table:**

N1—C1	1.336 (3)	C5—C5^i^	1.490 (4)
N1—C5	1.348 (3)	C6—H6A	0.9900
N2—C7	1.329 (3)	C6—H6B	0.9900
N2—C8	1.361 (3)	C7—H7	0.9500
N2—C6	1.474 (3)	C8—C9	1.354 (3)
N3—C7	1.324 (3)	C8—H8	0.9500
N3—C9	1.370 (3)	C9—H9	0.9500
N3—C10	1.466 (3)	C10—H10A	0.9800
C1—C2	1.385 (3)	C10—H10B	0.9800
C1—H1	0.9500	C10—H10C	0.9800
C2—C3	1.380 (3)	P1—F5	1.5869 (16)
C2—C6	1.513 (3)	P1—F3	1.5872 (17)
C3—C4	1.388 (3)	P1—F6	1.5899 (18)
C3—H3	0.9500	P1—F2	1.5947 (16)
C4—C5	1.386 (3)	P1—F4	1.6014 (16)
C4—H4	0.9500	P1—F1	1.6069 (15)
			
C1—N1—C5	117.10 (19)	N3—C7—H7	125.4
C7—N2—C8	108.3 (2)	N2—C7—H7	125.4
C7—N2—C6	124.6 (2)	C9—C8—N2	107.4 (2)
C8—N2—C6	127.2 (2)	C9—C8—H8	126.3
C7—N3—C9	108.2 (2)	N2—C8—H8	126.3
C7—N3—C10	124.8 (2)	C8—C9—N3	107.0 (2)
C9—N3—C10	126.9 (2)	C8—C9—H9	126.5
N1—C1—C2	124.9 (2)	N3—C9—H9	126.5
N1—C1—H1	117.5	N3—C10—H10A	109.5
C2—C1—H1	117.5	N3—C10—H10B	109.5
C3—C2—C1	117.3 (2)	H10A—C10—H10B	109.5
C3—C2—C6	124.1 (2)	N3—C10—H10C	109.5
C1—C2—C6	118.6 (2)	H10A—C10—H10C	109.5
C2—C3—C4	119.2 (2)	H10B—C10—H10C	109.5
C2—C3—H3	120.4	F5—P1—F3	90.25 (10)
C4—C3—H3	120.4	F5—P1—F6	91.05 (10)
C5—C4—C3	119.5 (2)	F3—P1—F6	178.66 (10)
C5—C4—H4	120.2	F5—P1—F2	91.12 (9)
C3—C4—H4	120.2	F3—P1—F2	89.70 (9)
N1—C5—C4	121.95 (19)	F6—P1—F2	90.60 (9)
N1—C5—C5^i^	116.7 (2)	F5—P1—F4	90.18 (9)
C4—C5—C5^i^	121.4 (2)	F3—P1—F4	90.27 (9)
N2—C6—C2	113.67 (19)	F6—P1—F4	89.41 (10)
N2—C6—H6A	108.8	F2—P1—F4	178.70 (9)
C2—C6—H6A	108.8	F5—P1—F1	179.80 (10)
N2—C6—H6B	108.8	F3—P1—F1	89.85 (9)
C2—C6—H6B	108.8	F6—P1—F1	88.84 (9)
H6A—C6—H6B	107.7	F2—P1—F1	89.04 (9)
N3—C7—N2	109.1 (2)	F4—P1—F1	89.66 (8)
			
C5—N1—C1—C2	−0.4 (4)	C3—C2—C6—N2	24.8 (3)
N1—C1—C2—C3	1.9 (4)	C1—C2—C6—N2	−157.2 (2)
N1—C1—C2—C6	−176.2 (2)	C9—N3—C7—N2	0.4 (3)
C1—C2—C3—C4	−0.9 (3)	C10—N3—C7—N2	−179.8 (2)
C6—C2—C3—C4	177.2 (2)	C8—N2—C7—N3	−0.2 (3)
C2—C3—C4—C5	−1.5 (3)	C6—N2—C7—N3	179.07 (19)
C1—N1—C5—C4	−2.2 (3)	C7—N2—C8—C9	0.0 (3)
C1—N1—C5—C5^i^	178.7 (3)	C6—N2—C8—C9	−179.3 (2)
C3—C4—C5—N1	3.1 (3)	N2—C8—C9—N3	0.2 (3)
C3—C4—C5—C5^i^	−177.8 (3)	C7—N3—C9—C8	−0.3 (3)
C7—N2—C6—C2	−88.2 (3)	C10—N3—C9—C8	179.8 (2)
C8—N2—C6—C2	91.0 (3)		

Symmetry codes: (i) −*x*+1, −*y*+2, −*z*+1.

### Hydrogen-bond geometry (Å, °)

**Table:**

*D*—H···*A*	*D*—H	H···*A*	*D*···*A*	*D*—H···*A*
C7—H7···F1	0.95	2.23	3.111 (3)	154.
C7—H7···F4	0.95	2.39	3.230 (3)	147.
C8—H8···F1^ii^	0.95	2.50	3.163 (3)	127.
C8—H8···F2^ii^	0.95	2.50	3.446 (3)	176.
C9—H9···F2^iii^	0.95	2.52	3.240 (3)	133.

Symmetry codes: (ii) −*x*+1, *y*+1/2, −*z*+1/2; (iii) *x*, −*y*+3/2, *z*−1/2.
